# Short-time quantum propagator and Bohmian trajectories^☆^

**DOI:** 10.1016/j.physleta.2013.08.031

**Published:** 2013-12-06

**Authors:** Maurice de Gosson, Basil Hiley

**Affiliations:** a Universität Wien, Fakultät für Mathematik, NuHAG, Wien 1090, Austria; b University of London, Birkbeck College, Theoretical Physics Unit, London WC1E 7HX, United Kingdom

**Keywords:** Generating function, Short-time action, Quantum potential, Bohmian trajectories

## Abstract

We begin by giving correct expressions for the short-time action following the work Makri–Miller. We use these estimates to derive an accurate expression modulo $\Delta t^{2}$ for the quantum propagator and we show that the quantum potential is negligible modulo $\Delta t^{2}$ for a point source, thus justifying an unfortunately largely ignored observation of Holland made twenty years ago. We finally prove that this implies that the quantum motion is classical for very short times.

## Highlights

• Gives correct expressions for the short-time action; following the work of one of the authors and Makri and Miller. Usually ignored in the literature.
• Derives a correct expression modulo $\Delta t^{2}$ for the quantum propagator.
• Shows that the quantum potential is negligible modulo $\Delta t^{2}$ for a point source.
• Shows that this implies that the quantum motion is classical for very short times.

## 1 Introduction

In exploring the WKB limit of quantum theory, Bohm [2] was the first to notice that although one starts with all the ambiguities about the nature of a quantum system, the first order approximation fits the ordinary classical ontology. By that we mean that the real part of the Schrödinger equation under polar decomposition of the wave function becomes the classical Hamilton–Jacobi equation in the limit where terms involving *ℏ* are neglected. In contrast to this approach, in this Letter we show that the classical trajectories arise from a short-time quantum propagator when terms of $O(\Delta t^{2})$ can be neglected. This fact was actually already observed by Holland some twenty years ago: In page 269 of his book [6] infinitesimal time intervals are considered whose sequence constructs a finite path. It is shown that along each segment the motion is classical (negligible quantum potential), and that it follows that the quantum path may be decomposed into a sequence of segments along each of which the classical action is a minimum. The novel contribution of the present Letter is an improved proof of Hollandʼs result using an improved version of the propagator due to Makri and Miller [9,10]. (See also de Gosson [3] for a further discussion.)

Now it is well known that explicit approximate expressions for the short-time action already play an essential role in various aspects of quantum mechanics (for instance the Feynman path integral, or semi-classical mechanics), and so does the associated Van Vleck determinant. Unfortunately, as already observed by Makri and Miller [9,10], these expressions, while giving the correct results for long time behavior, are not accurate enough to allow us to explore the short-time propagator rigorously. It is actually worse, the literature seems to be dominated by formulas which Makri and Miller show are wrong even to the first order of approximation!

These results have enabled us to provide precise estimates for the short-time Bohmian quantum trajectories for an initially sharply located particle. We will see that these trajectories are classical to the second order in time, due to the vanishing of the quantum potential for small time intervals.

In this Letter we sidestep the philosophical and ontological debate around the “reality” of Bohmʼs trajectories and rather focus on the mathematical issues.

## 2 Bohmian trajectories

Consider a time-dependent Hamiltonian function

(1) $$H(x,p,t)=\sum_{j=1}^{n}\frac{p_{j}^{2}}{2m_{j}}+U(x,t)$$

and the corresponding quantum operator

(2) $$\hat{H}(x,-i\hbar \nabla_{x},t)=\sum_{j=1}^{n}\frac{-\hbar^{2}}{2m_{j}}\frac{\partial^{2}}{\partial x_{j}^{2}}+U(x,t).$$

The associated Schrödinger equation is

(3) $$i\hbar \frac{\partial \Psi }{\partial t}=\hat{H}(x,-i\hbar \nabla_{x},t)\Psi ,\;\Psi (x,0)=\Psi_{0}(x).$$

Let us write *Ψ* in polar form $Re^{iS/\hbar }$; here R=R(x,t)⩾0 and S=S(x,t) are real functions. On inserting $Re^{iS/\hbar }$ into Schrödingerʼs equation and separating real and imaginary parts, one sees that the functions *R* and *S* satisfy, at the points (x,t) where R(x,t)>0, the coupled system of non-linear partial differential equations

(4) $$\frac{\partial S}{\partial t}+\sum_{j=1}^{n}\frac{1}{2m_{j}}{\left(\frac{\partial S}{\partial x_{j}}\right)}^{2}+U(x,t)-\sum_{j=1}^{n}\frac{\hbar^{2}}{2m_{j}R}\frac{\partial^{2}R}{\partial x_{j}^{2}}=0,$$

(5) $$\frac{\partial R^{2}}{\partial t}+\sum_{j=1}^{n}\frac{1}{m_{j}}\frac{\partial }{\partial x_{j}}\left(R^{2}\frac{\partial S}{\partial x_{j}}\right)=0.$$

The crucial step now consists in recognizing the first equation as a Hamilton–Jacobi equation, and the second as a continuity equation. In fact, introducing the quantum potential

(6) $$Q^{\Psi }=-\sum_{j=1}^{n}\frac{\hbar^{2}}{2m_{j}R}\frac{\partial^{2}R}{\partial x_{j}^{2}}$$

(Bohm and Hiley [2]) and the velocity field

(7) $$v^{\Psi }(x,t)=\left(\frac{1}{m_{1}}\frac{\partial S}{\partial x_{1}},\ldots ,\frac{1}{m_{n}}\frac{\partial S}{\partial x_{n}}\right)$$

Eqs. (4) and (5) become

(8) $$\frac{\partial S}{\partial t}+H(x,\nabla_{x}S,t)+Q^{\Psi }(x,t)=0,$$

(9) $$\frac{\partial \rho }{\partial t}+\mathrm{div}\left(\rho v^{\Psi }\right)=0,\;\rho =R^{2}.$$

The main postulate of the Bohmian theory of motion is that particles follow quantum trajectories, and that these trajectories are the solutions of the differential equations

(10) x˙jΨ=ℏmjIm1Ψ∂Ψ∂xj.

The phase space interpretation is that the Bohmian trajectories are determined by the equations

(11) $${\dot{x}}_{j}^{\Psi }=\frac{1}{m_{j}}p_{j}^{\Psi },\;{\dot{p}}_{j}^{\Psi }=-\frac{\partial U}{\partial x_{j}}\left(x^{\Psi },t\right)-\frac{\partial Q^{\Psi }}{\partial x_{j}}\left(x^{\Psi },t\right).$$

It is straightforward to check that these are just Hamiltonʼs equations for the Hamiltonian function

(12) $$H^{\Psi }(x,p,t)=\sum_{j=1}^{n}\frac{p_{j}^{2}}{2m_{j}}+U(x,t)+Q^{\Psi }(x,t)$$

which can be viewed as a perturbation of the original Hamiltonian *H* by the quantum potential $Q^{\Psi }$ (see Holland [7,8] for a detailed study of quantum trajectories in the context of Hamiltonian mechanics).

The Bohmian equations of motion are *a priori* only defined when R≠0 (that is, outside the nodes of the wave function); this will be the case in our constructions since for sufficiently small times this condition will be satisfied by continuity if we assume that it is case at the initial time.

An important feature of the quantum trajectories defined above is that they cannot cross; thus there will be no conjugate points like those that complicate the usual Hamiltonian dynamics.

## 3 The short-time propagator

The solution *Ψ* of Schrödingerʼs equation (3) can be written

(13) $$\Psi (x,t)=\int K(x,x_{0};t)\Psi_{0}(x_{0})\;dx_{0}$$

where the kernel *K* is the quantum propagator:

(14) $$K(x,x_{0};t)=\langle x|\exp(-i\hat{H}t/\hbar )|x_{0}\rangle .$$

Schrödingerʼs equation (3) is then equivalent to

(15) $$i\hbar \frac{\partial K}{\partial t}=\hat{H}(x,-i\hbar \nabla_{x},t)K,\;K(x,x_{0};0)=\delta (x-x_{0})$$

where *δ* is the Dirac distribution. Physically this equation describes an isotropic source of point-like particle emanating from the point $x_{0}$ at initial time $t_{0}=0$. We want to find an asymptotic formula for *K* for short time intervals Δ*t*. Referring to the usual literature (see, e.g., Schulman [11]), such approximations are given by expressions of the type

$$K(x,x_{0};\Delta t)={\left(\frac{1}{2\pi i\hbar }\right)}^{n/2}\sqrt{\rho (x,x_{0};\Delta t)}\exp\left(\frac{i}{\hbar }S(x,x_{0};\Delta t)\right)$$

where $S(x,x_{0};\Delta t)$ is the action along the classical trajectory from $x_{0}$ to *x* in time Δ*t* and

$$\rho (x,x_{0};\Delta t)=\det{\left(-\frac{\partial^{2}S(x,x_{0};\Delta t)}{\partial x_{j}\partial x_{k}}\right)}_{1⩽j,k⩽n}$$

is the corresponding Van Vleck determinant. We will need precise short-time behavior of the action. In this regard Makri and Miller [9,10] have shown that the asymptotic expression for the generating function is given by

(16) $$S(x,x_{0};\Delta t)=\sum_{j=1}^{n}\frac{m_{j}}{2\Delta t}{\left(x_{j}-x_{0}\right)}^{2}-\tilde{U}(x,x_{0})\Delta t+O\left(\Delta t^{2}\right)$$

where $\tilde{U}(x,x_{0},0)$ is the average value of the potential over the straight line joining $x_{0}$ at time $t_{0}$ to *x* at time *t* with constant velocity:

(17) $$\tilde{U}(x,x_{0})=\int_{0}^{1}U\left(\lambda x+(1-\lambda )x_{0},0\right)\;d\lambda .$$

For instance when

$$H(x,p)=\frac{1}{2m}\left(p^{2}+m^{2}\omega^{2}x^{2}\right)$$

is the one-dimensional harmonic oscillator formula (16) yields the correct expansion

(18) $$S(x,x_{0};t)=\frac{m}{2\Delta t}{\left(x-x_{0}\right)}^{2}-\frac{m\omega^{2}}{6}\left(x^{2}+xx_{0}+x_{0}^{2}\right)\Delta t+O\left(\Delta t^{2}\right),$$

the latter can of course be deduced directly from the exact value

(19) $$S(x,x_{0};t,t_{0})=\frac{m\omega }{2\sin\omega \Delta t}\left(\left(x^{2}+x_{0}^{2}\right)\cos\omega \Delta t-2xx_{0}\right)$$

by expanding sinωΔt and cosωΔt for Δt→0.

Introducing the following notation,

(20) $$\tilde{S}(x,x_{0};\Delta t)=\sum_{j=1}^{n}m_{j}\frac{{\left(x_{j}-x_{0}\right)}^{2}}{2\Delta t}-\tilde{U}(x,x_{0})\Delta t,$$

leads us to the Makri and Miller approximation (formula (17c) in [9]) for the short-time propagator:

(21) $$K(x,x_{0};\Delta t)={\left(\frac{1}{2\pi i\hbar }\right)}^{n/2}\sqrt{\rho (x,x_{0};\Delta t)}\exp\left(\frac{i}{\hbar }\tilde{S}(x,x_{0};\Delta t)\right)+O\left(\Delta t^{2}\right)$$

where

$$\rho (x,x_{0};\Delta t)=\det{\left(-\frac{\partial^{2}\tilde{S}(x,x_{0};\Delta t)}{\partial x_{j}\partial x_{0,k}}\right)}_{1⩽j,k⩽n}.$$

It turns out that this formula can be somewhat improved. The Van Vleck determinant $\rho (x,x_{0};\Delta t)$ is explicitly given, taking formula (20) into account, by

$$\rho (x,x_{0};\Delta t)=\det\left(-\frac{1}{\Delta t}M-{\tilde{U}}_{x,x_{0}}^{\prime \prime }(x,x_{0})\Delta t\right)$$

where *M* is the mass matrix (the diagonal matrix with positive entries the masses $m_{j}$) and

$${\tilde{U}}_{x,x_{0}}^{\prime \prime }={\left(-\frac{\partial^{2}\tilde{U}(x,x_{0})}{\partial x_{j}\partial x_{k}}\right)}_{1⩽j,k⩽n}.$$

Writing

$$\left(-\frac{1}{\Delta t}M-{\tilde{U}}_{x,x_{0}}^{\prime \prime }(x,x_{0})\Delta t\right)=-\frac{1}{\Delta t}M\left[I_{n\times n}-M^{-1}{\tilde{U}}_{x,x_{0}}^{\prime \prime }(x,x_{0})\Delta t^{2}\right]=-\frac{1}{\Delta t}M\left[I_{n\times n}+O\left(\Delta t^{2}\right)\right],$$

we have by taking the determinant of both sides

$$\rho (x,x_{0};\Delta t)=\frac{m_{1}\cdot m_{n}}{{\left(\Delta t\right)}^{n}}\det\left(I_{n\times n}+O\left(\Delta t^{2}\right)\right).$$

Noting that $\det(I_{n\times n}+O(\Delta t^{2}))=1+O(\Delta t^{2})$, we thus have

(22) $$\rho (x,x_{0};\Delta t)=\frac{m_{1}\cdots m_{n}}{{\left(\Delta t\right)}^{n}}\left(1+O\left(\Delta t^{2}\right)\right).$$

Writing

(23) $$\tilde{\rho }(\Delta t)=\frac{m_{1}\cdots m_{n}}{{\left(\Delta t\right)}^{n}}$$

which is just the Van Vleck density for the free particle Hamiltonian. We thus have

(24) $$\rho (x,x_{0};\Delta t)=\tilde{\rho }(\Delta t)\left(1+O\left(\Delta t^{2}\right)\right)$$

and hence we can rewrite formula (21) as

(25) $$K(x,x_{0};\Delta t)={\left(\frac{1}{2\pi i\hbar }\right)}^{n/2}\sqrt{\tilde{\rho }(\Delta t)}\exp\left(\frac{i}{\hbar }\tilde{S}(x,x_{0};\Delta t)\right)+O\left(\Delta t^{2}\right).$$

We will see below that this formula allows an easy study of the quantum potential for *K*.

## 4 Short-time Bohmian trajectories

Let us determine the quantum potential *Q* corresponding to the propagator $K=K(x,x_{0};t)$ using the asymptotic formulas above. Recall that it describes an isotropic source of point-like particle emanating from the point $x_{0}$ at initial time $t_{0}=0$. We have, by definition,

$$Q=-\sum_{j=1}^{n}\frac{\hbar^{2}}{2m_{j}\sqrt{\rho }}\frac{\partial^{2}\sqrt{\rho }}{\partial x_{j}^{2}}$$

which we can rewrite

$$Q=-{\frac{\hbar }{2}}^{2}\frac{M^{-1}\nabla_{x}\cdot \nabla_{x}\sqrt{\rho }}{\sqrt{\rho }}$$

where *M* is the mass matrix defined above. We have, using (24),

$$\sqrt{\rho }=\sqrt{\tilde{\rho }(\Delta t)}\left(1+O\left(\Delta t^{2}\right)\right)$$

and hence

$$\frac{\partial^{2}\sqrt{\rho }}{\partial x_{j}^{2}}=O\left({\left(\Delta t\right)}^{2}\right)).$$

From this it follows that the quantum potential associated with the propagator satisfies

(26) $$Q(x,x_{0};\Delta t)=O\left(\Delta t^{2}\right).$$

The discussion above suggests that the quantum trajectory of a sharply located particle should be identical with the classical (Hamiltonian) trajectory for short times. Let us show this is indeed the case. If we want to monitor the motion of such a particle, we have of course to specify its initial momentum which gives its direction of propagation at time $t_{0}=0$; we set

(27) $$p(0)=p_{0}.$$

In view of formula (10), the trajectory in position space is obtained by solving the system of differential equations

(28) x˙=ℏImM−1∇xKK,x(0)=x0.

Replacing *K* with its approximation

$$\tilde{K}(x,x_{0};\Delta t)={\left(\frac{1}{2\pi i\hbar }\right)}^{n/2}\sqrt{\tilde{\rho }(\Delta t)}\exp\left(\frac{i}{\hbar }\tilde{S}(x,x_{0};\Delta t)\right)$$

we have, since $K-\tilde{K}=O(\Delta t^{2})$ in view of (25),

x˙=ℏImM−1∇xK˜K˜+O(Δt2).

A straightforward calculation, using the expression (20) for the approximate action $\tilde{S}(x,x_{0};\Delta t)$, leads to the equation

(29) $$\dot{x}(\Delta t)=\frac{x(\Delta t)-x_{0}}{\Delta t}-M^{-1}\nabla_{x}\tilde{U}\left(x(\Delta t),x_{0}\right)\Delta t+O\left(\Delta t^{2}\right)$$

(*cf*. the proof of Lemma 248 in [3]). This equation is singular at time t=0 hence the initial condition $x(0)=x_{0}$ is not sufficient for finding a unique solution; this is of course consistent with the fact that (29) describes an arbitrary particle emanating from $x_{0}$; to single out one quantum trajectory we have to use the additional condition (27) giving the direction of the particle at time t=0 (see the discussion in Holland (1993) [6, §6.9]). We thus have

$$x(\Delta t)=x_{0}+M^{-1}p_{0}\Delta t+O\left(\Delta t^{2}\right);$$

in particular $x(\Delta t)=x_{0}+O(\Delta t)$ and hence, by continuity,

$$\nabla_{x}\tilde{U}\left(x(\Delta t),x_{0}\right)=\nabla_{x}\tilde{U}(x_{0},x_{0})+O(\Delta t).$$

Let us calculate $\nabla_{x}\tilde{U}(x_{0},x_{0})$. We have, taking definition (17) into account,

$$\nabla_{x}\tilde{U}(x,x_{0})=\int_{0}^{1}\lambda \nabla_{x}U\left(\lambda x+(1-\lambda )x_{0},0\right)\;d\lambda$$

and hence

$$\nabla_{x}\tilde{U}(x_{0},x_{0})=\int_{0}^{1}\lambda \nabla_{x}U(x_{0},0)\;d\lambda =\frac{1}{2}\nabla_{x}U(x_{0},0).$$

We can thus rewrite Eq. (29) as

$$\dot{x}(\Delta t)=\frac{x(\Delta t)-x_{0}}{\Delta t}-\frac{1}{2}M^{-1}\nabla_{x}U(x_{0},0)\Delta t+O\left(\Delta t^{2}\right).$$

Let us now differentiate both sides of this equation with respect to Δ*t*:

(30) $$\ddot{x}(t)=\frac{x(t)-x_{0}}{{\left(\Delta t\right)}^{2}}+\frac{\dot{x}(t)}{\Delta t}-\frac{1}{2}M^{-1}\nabla_{x}U(x_{0},0)+O(\Delta t)$$

that is, replacing $\dot{x}(\Delta t)$ by the value given by (29),

(31) $$\dot{p}(t)=M\ddot{x}(t)=-\nabla_{x}U(x_{0},0)+O(\Delta t).$$

Solving this equation we get

(32) $$p(t)=p_{0}-\nabla_{x}U(x_{0},0)\Delta t+O\left(\Delta t^{2}\right).$$

Summarizing, the solutions of the Hamilton equations are given by

(33) $$x(\Delta t)=x_{0}+\frac{p_{0}}{m}\Delta t+O\left(\Delta t^{2}\right),$$

(34) $$p(\Delta t)=p_{0}-\nabla_{x}U(x_{0},0)\Delta t+O\left(\Delta t^{2}\right).$$

These equations are, up to the error terms $O(\Delta t^{2})$ the equations of motion of a classical particle moving under the influence of the potential *U*; there is no trace of the quantum potential, which is being absorbed by the terms $O(\Delta t^{2})$. The motion is thus identical with the classical motion on time scales of order $\Delta t^{2}$.

## 5 Conclusion

This result puts Bohmʼs original perception, which led him to the causal interpretation, on a firm mathematical footing. He writes [1]

Indeed it had long been known that when one makes a certain approximation (WKB) Schrödingerʼs equation becomes equivalent to the classical Hamilton–Jacobi theory. At a certain point I asked myself: What would happen, in the demonstration of this equivalence, if we did not make this approximation? I saw immediately that there would be an additional potential, representing a kind of force, that would be acting on the particle.

The source of this “force” was the quantum potential. In our approach we see that while any classical potential acts immediately, the quantum potential does not. From this fact two consequences follow.

Firstly, it provides a rigorous treatment of the “watched pot” effect. If we keep observing a particle that, if unwatched would make a transition from one quantum state to another, will now no longer make that transition. The unwatched transition occurs when the quantum potential grows to produce the transition. Continuously observing the particle does not allow the quantum potential to develop so the transition does not take place. We will not discuss this effect further here as it has been reported in detail in [4]. For an other application see Section 6.9 in Bohm and Hiley [2].

Secondly, in the situation when the quantum potential decreases continuously with time, the quantum trajectory continuously deforms into a classical trajectory [5]. This means that there is no need to appeal to decoherence to reach the classical domain.
